# Invasive splenic mucormycosis due to *Rhizopus microsporus* during chemotherapy for acute monocytic leukemia: a case report and literature review

**DOI:** 10.3389/fonc.2023.1237807

**Published:** 2023-09-08

**Authors:** Xiru Peng, Zixiu Wei, Lijuan Wang, Juan Cheng

**Affiliations:** ^1^ The First Clinical Medical College of Lanzhou University, Lanzhou, Gansu, China; ^2^ Department of Hematology, The First Hospital of Lanzhou University, Lanzhou, China

**Keywords:** invasive splenic mucormycosis, *Rhizopus microsporus*, metagenomic next-generation sequencing, contrast-enhanced ultrasonography, amphotericin B

## Abstract

Mucormycosis is a rare opportunistic fungal infection associated with high mortality that typically occurs in immunocompromised patients. It is difficult to diagnose owing to non-specific clinical manifestations, the serologic index, imaging features, and the limitations of diagnostic methods. The incidence of invasive splenic mucormycosis is extremely rare, with only a few cases documented in the literature. We report a survival case of invasive splenic mucormycosis involving the liver caused by *Rhizopus microsporus* in a patient during consolidation therapy for acute monocytic leukemia (AML-M_5_). The patient initially presented with recurrent fever and splenomegaly accompanied by multiple focal hypodensities unresponsive to empiric anti-infective treatment. Splenic mucormycosis was diagnosed by Contrast-Enhanced Ultrasonography (CEUS) and metagenomic next-generation sequencing (mNGS). However, surgical intervention carries a high risk due to the progressive involvement of the liver in invasive splenic mucormycosis. Fortunately, monotherapy with amphotericin B was effective, and the patient underwent allo-HSCT. This case aims to emphasize the importance of utilizing mNGS and CEUS for the timely diagnosis of mucormycosis to help clinicians identify splenic mucormycosis and initiate appropriate therapy as soon as possible to improve therapeutic efficacy and prognosis.

## Introduction

Mucormycosis is a rare but life-threatening opportunistic fungal infection associated with immunocompromised patients with hematologic malignancies, persistent neutropenia, poorly controlled diabetes mellitus, iron overload, and trauma, undergoing solid organ or hematopoietic stem cell transplantation (HSCT), or receiving corticosteroid therapy (1). The reported incidence varies from 0.005 to 1.7 per million people, depending on geographic region and study center (2). Mortality rates vary between 40% and 80%, depending on the location and extent of the lesions and the severity of the patient’s primary disease (3). The most frequently affected sites of mucormycosis are pulmonary, rhino-orbital-cerebral, and cutaneous, while invasive splenic mucormycosis is extremely rare, with only a handful of cases documented in the literature. The recommended initial therapy involves liposomal amphotericin B and surgical debridement (4). However, the clinical manifestations of splenic mucormycosis are non-specific, leading to frequent delays in diagnosis. Early identification and prompt initiation of targeted antifungal therapy are essential to improving the patient’s prognosis.

Here, we report a survival case of invasive splenic mucormycosis involving the liver caused by *Rhizopus microsporus* in a patient during consolidation therapy for acute monocytic leukemia (AML-M_5_). The patient initially presented with recurrent fever and splenomegaly accompanied by multiple focal hypodensities unresponsive to empiric anti-infective treatment. Subsequently, splenic mucormycosis was diagnosed by Contrast-Enhanced Ultrasonography (CEUS) and metagenomic next-generation sequencing (mNGS). However, surgical intervention carries a high risk due to the progressive involvement of the liver in invasive splenic mucormycosis. Fortunately, after treatment with amphotericin B as an antifungal, the patient’s symptoms, inflammatory markers, and imaging results showed significant improvement. Subsequently, allo-HSCT was performed on 15/06/2023. During the transplantation process, prophylactic anti-mucormycosis therapy with amphotericin B was continued, and the patient achieved hematopoietic reconstruction. The aim of this case is to emphasize the importance of utilizing mNGS and CEUS in the diagnosis of mucormycosis to help clinicians identify splenic mucormycosis and initiate appropriate therapy as soon as possible to improve therapeutic efficacy and prognosis.

## Case report

**Figure 1:**
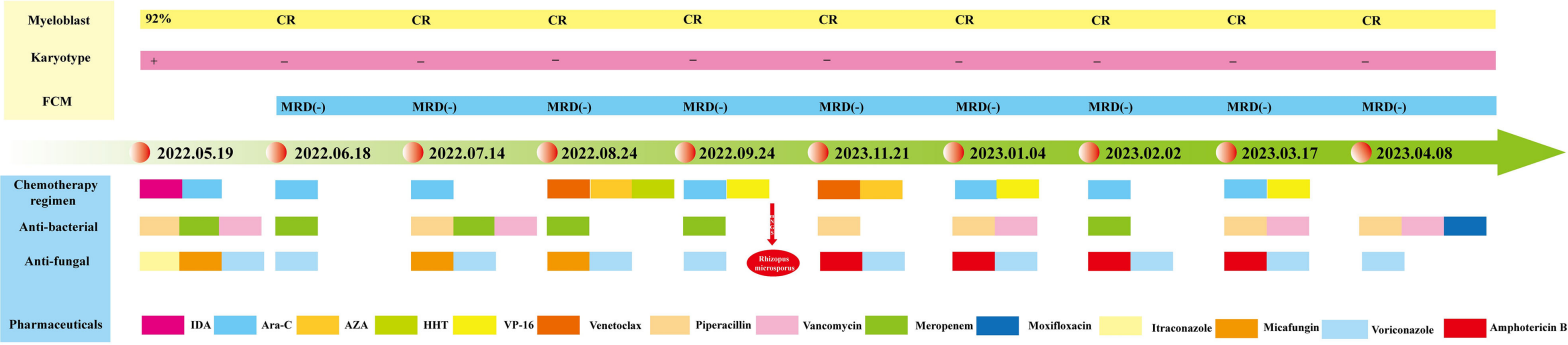
Timeline of the patient’s diagnosis, treatment, and evaluation of clinical efficacy. The patient achieved complete morphologic and genetic remission after induction therapy with idarubicin and cytarabine. During the subsequent consolidation therapy, the patient maintained a continuous complete remission, and the diagram illustrates in detail the therapeutic protocol for anti-infective treatment.

A 24-year-old female patient was admitted on 10/05/2022, for “recurrent headache, pharyngalgia and fever for 1 week”. The patient denied any history of hypertension, diabetes, coronary heart disease, or infectious diseases such as hepatitis B and tuberculosis. Physical examination on admission showed mucocutaneous pallor, and palpation did not detect superficial lymph node enlargement or hepatosplenomegaly. Laboratory analyses revealed pancytopenia: white blood cell (WBC) count 1.37×10^9^/L, red blood cell (RBC) count 2.3×10^12^/L, platelet (PLT) count 71×10^9^/L, absolute neutrophil count (ANC) 0.3×10^9^/L, and hemoglobin (Hb) count 69 g/L. Abdominal ultrasonography and computed tomography (CT) scans showed no abnormalities. The patient was diagnosed with AML-M_5_ on 19/05/2022, based on a comprehensive assessment of morphology, immunophenotype, cytogenetics, and molecular biology (MICM). She had achieved a complete cytogenetic response, and minimal residual disease (MRD) was negative by flow cytometry after starting a cycle of induction chemotherapy with idarubicin and cytarabine. She then received consolidation therapy with high-dose cytarabine, VAH (venetoclax+azacitidine+homoharringtonine), AE (cytarabine+etoposide), and VA (venetoclax+azacitidine). A treatment timeline is shown in **Figure 1**.

However, during the third cycle of consolidation therapy, the patient developed a recurrent fever and intermittent discomfort in the left upper quadrant. The laboratory analysis results were as follows: WBC 0.82×10^9^/L, RBC 2.1×10^12^/L, PLT 90×10^9^/L, ANC 0.4×10^9^/L, and Hb 59 g/L. C-reactive protein (78.81 mg/L) and procalcitonin (0.11 ng/mL) were both elevated. Abdominal ultrasonography and CT scans revealed splenomegaly with multiple focal hypodensities, while no evident abnormalities were detected in the liver, gallbladder, pancreas, or kidney, which initially suggested leukemic involvement of the spleen. However, despite empiric treatment with piperacillin sodium and tazobactam sodium, meropenem, vancomycin, voriconazole, and micafungin, satisfactory therapeutic effects were not obtained. The patient exhibited recurrent fever, and the abdominal CT scan showed progression of the splenic lesions to the liver. To further clarify the nature of the splenic lesions, we performed splenic CEUS, which revealed multiple hypoechoic nodules in the spleen that were considered splenic abscesses. The etiology of these infections may be related to the primary disease or myelosuppression after chemotherapy. Notably, no pathogens were detected in repeated blood cultures, and both galactomannan and serum (1, 3)-β-D-glucan tests yielded negative results. Fortunately, *Rhizopus microsporus* was identified by peripheral blood mNGS, providing a basis for targeted anti-fungal therapy. The recommended therapeutic approach for mucormycosis involves a combination of anti-fungal pharmacotherapy and surgical debridement of necrotic tissue. However, the follow-up CT scan showed that the splenic mucormycosis had progressed to the liver, resulting in multiple focal hypodensities in both organs. Considering the multiple infected lesions and deteriorating systemic condition, surgical intervention was not an optimal option in this particular case.

**Figure 2:**
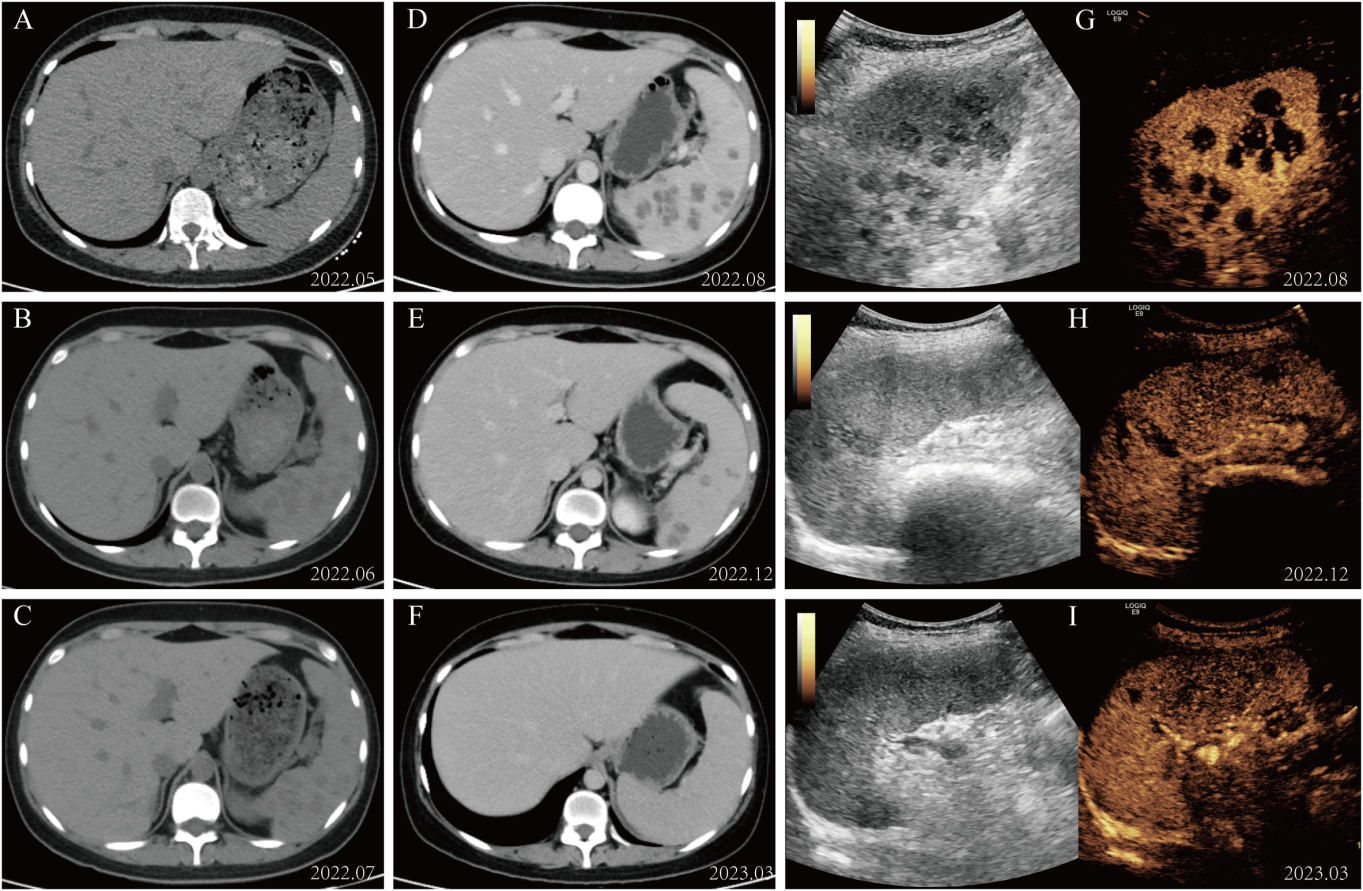
Imaging alterations during patient management. **(A–C)** The abdominal CT scan revealed diffuse small round low-density lesions in the spleen. **(D–F)** The splenic lesions exhibited significant resolution following treatment with amphotericin B. **(G–I)** Changes in contrast-enhanced ultrasound of the spleen during administration of amphotericin B therapy.

Based on these results, we initiated intravenous pumping of amphotericin B cholesteryl sulfate complex (1 mg/kg/day) for hepatosplenic mucormycosis. Based on therapeutic efficacy and adverse reactions, the dosage was gradually increased to 3 mg/kg/day from December 2022 to March 2023. The subsequent abdominal CT scan and splenic CEUS showed a gradual resolution of hepatic and splenic hypodensities. After approximately 4 months of treatment with liposomal amphotericin B, with a cumulative dose of 8.1 g administered, the patient was well and asymptomatic, and the lesions on imaging examinations improved considerably. Fortunately, in our study, the patient did not exhibit any signs of renal dysfunction but solely presented with hypokalemia, which was cured after potassium supplementation. The patient underwent allo-HSCT and received prophylactic antifungal treatment with liposomal amphotericin B due to a previous mucormycosis infection. The imaging results of the patient throughout the course of treatment are shown in **Figure 2**.

## Discussion

Mucormycosis is caused by opportunistic pathogenic fungi of the order Mucorales, which includes *Rhizopus*, *Rhizomucor*, *Mucor*, *Cunninghamella*, *Lichtheimia*, *Saksenaea*, and *Apophysomyces*. These are saprophytic fungi that are widespread in our environment, usually found in soil and decaying matter, with broad, ribbon-like aseptate hyphae that characteristically branch at right angles (5). The fungus is spread by spores of the order Mucorales, particularly through food contamination, inhalation, or infection of open wounds (5). The incidence and prevalence of mucormycosis exhibit regional variations across different countries. Globally, the highest reported incidence of mucormycosis comes from India (6). Osaigbovo et al. have reported 408 individual cases from 12 African countries: 80.9% from North Africa, 15.4% from Southern Africa, 1.7% from East Africa, 1.7% from West Africa, and 0.2% from Central Africa (7). The average incidence of mucormycosis in the USA is about 0.3 cases per 100,000 per year, compared to 0.54 cases per million people in Greece. In mainland China, 390 cases of mucormycosis were reported in two decades, from 2001 to 2020. It is noteworthy that the incidence of mucormycosis has increased dramatically worldwide since the onset of COVID-19 (8). Diabetes mellitus, malignancy, solid organ or HSCT, severe neutropenia, immunosuppressive therapy, iron overload, malnutrition, and COVID-19 are significant risk factors of mucormycosis (9). It is imperative for clinicians to be aware that mucormycosis may occur in patients with high-risk factors, especially when conventional anti-infective therapy proves ineffective. The prognosis of mucormycosis remains poor, particularly in patients with hematologic malignancies. In medical practice, the index of suspicion for mucormycosis should be increased. Timely diagnosis and appropriate treatment are critical to reducing the mortality of mucormycosis.

Based on anatomical localization, mucormycosis can be classified into six forms: rhino-orbital-cerebral, cutaneous, pulmonary, gastrointestinal, disseminated, and uncommon sites (10). Due to its diverse and atypical clinical manifestations, early diagnosis of mucormycosis is challenging. The incidence of invasive splenic mucormycosis is extremely rare, with only a few cases documented in the medical literature. Hammami et al. conducted a comprehensive review of all reported cases of splenic mucormycosis up until October 2020, revealing that the clinical manifestations of this condition primarily include fever, chills, abdominal pain, diarrhea, cough, dyspnea, loss of appetite, nausea, and vomiting, in addition to febrile neutropenia, depending on the organs affected. It can be inferred that mucormycosis lacks specific clinical features, and in the early stage, it may only present with symptoms such as fever and upper abdominal discomfort (11). In our case, the patient presented with recurrent fever and left upper quadrant discomfort, and imaging showed the development of multiple focal splenic hypodensities despite the administration of broad-spectrum antibiotics. When making a differential diagnosis, it is crucial to distinguish hepatosplenic mucormycosis, candidiasis, and leukemic infiltration. CEUS is an essential method for the diagnosis of focal hepatic and splenic lesions. Cao conducted a study on the role of contrast-enhanced ultrasound in differentiating benign from malignant splenic lesions and found that hypovascular enhancement was predominantly observed in malignancies, while a lack of enhancement was exclusively identified in benign cases (12). In our study, the splenic lesions had multiple focal hypodensities in abdominal ultrasonography, indicating leukemic involvement of the spleen. However, the splenic lesions did not show enhancement in the arterial and parenchymal phases on splenic CEUS, which is inconsistent with typical leukemic infiltration. In 2016, Trenker et al. reported the use of CEUS in the diagnosis of hepatosplenic mucormycosis in a patient with Chronic Lymphocytic Leukemia (13). To our knowledge, there is limited literature on the application of CEUS for the diagnosis of hepatosplenic mucormycosis. The lack of contrast enhancement on CEUS may help to distinguish invasive fungal infection from parenchymal leukemic infiltrates and the general need to confirm tumor progression seen radiologically by histopathology.

As the initial clinical symptoms and imaging findings of mucormycosis can be non-specific, diagnosis is often delayed, and misdiagnosis rates are high, leading to unsatisfactory treatment and high mortality rates. The gold standard for the diagnosis of mucormycosis is fungal culture and pathologic tissue biopsy. Fungal culture has high specificity, which is crucial for accurate species identification and anti-fungal susceptibility testing. However, its time-consuming nature and high false-negative rate limit its utility, with a sensitivity of only 25% (14). This can be attributed to several reasons, such as the grinding or homogenization of tissue specimens, which can destroy the delicate hyphae of mucor; the presence of genera that require special culture conditions; recent or ongoing therapy with an anti-fungal effective against Mucorales; or even a lack of expertise (6). Pathologic evaluation of biopsy specimens can provide a more definitive diagnosis, but obtaining tissue biopsies is not feasible for vulnerable AML patients (15). Molecular biology techniques can be utilized to diagnose mucormycosis with high speed and precision (16), owing to the fact that the amount of circulating Mucorales DNA is usually higher than that other fungal diseases due to the angio-invasive nature of Mucorales infection. Serum Mucorales qPCR is a non-invasive and extremely accurate diagnostic technique for mucormycosis. The results indicate that serum Mucorales PCR has a sensitivity of 85.2%, a specificity of 89.8%, and positive and negative likelihood ratios of 8.3 and 0.17, respectively, for diagnosing mucormycosis (17). However, PCR also has certain limitations: for instance, its specificity is dependent on primer design, and its sensitivity is prone to false-positive results after inspection contamination. In contrast, mNGS can detect almost all pathogenic bacteria in a sample. It does not require pre-guessing or hypotheses about the type of pathogen causing the infection (18). In addition, mNGS allows for efficient diagnosis of infectious diseases where culture may take a long time to grow or even produce false-negative results. mNGS is primarily used for pathogen screening, while PCR serves as a quantitative detection tool. Liu et al. reported a case of intracranial infection that did not respond to empiric antibiotic therapy and whose blood culture results were negative. However, NGS revealed an infection caused by Mucorales, and the fever resolved after targeted anti-fungal treatment with amphotericin B liposomes (15). Zhang et al. evaluated the diagnostic value of mNGS in 13 patients with hematologic malignancies suspected of mucormycosis, and their findings demonstrate that mNGS can be used not only for early diagnosis of mucormycosis, but also for simultaneous detection of bacteria and viruses, thereby improving the prognosis of patients (19). In our case, the predominant clinical manifestation was fever, and splenomegaly was revealed by abdominal ultrasonography and CT scans with multiple focal hypodensities. Although the results of blood cultures, serum (1, 3)-β-D-glucan, and galactomannan assays were negative, mNGS confirmed *Rhizopus microsporus* infection. After receiving timely treatment with intravenous pumping of amphotericin B cholesteryl sulfate complex, the patient’s symptoms resolved, and imaging examinations showed considerable improvement in the lesions. This suggests that the detection of Mucorales by mNGS in the blood may serve as an early marker for invasive splenic mucormycosis, potentially reducing treatment delay.

The main treatment options include anti-fungal therapy with amphotericin B, surgical resection, and the management of underlying risk factors (20). As established in all guidelines, surgical debridement is important in the control of mucormycosis, and several studies have confirmed the efficacy of surgical intervention in improving survival rates. However, it is also important to acknowledge that such procedures may also carry inherent risks, and selecting the safest treatment modality should be based on the patient’s clinical condition. In some cases, surgery may not be possible. Due to the presence of multiple lesions, surgical intervention was not a viable option in our particular case. Fortunately, the patient responded well to treatment with amphotericin B liposome alone and showed significant lesion improvement on imaging examinations. Posaconazole and isavuconazole may serve as alternative therapies or salvage prescriptions for patients who are unresponsive to or intolerant to conventional treatments (21). Clinical registries of the European Confederation of Medical Mycology have reported a success rate of approximately 50%-60% for first-line treatment with posaconazole in patients with mucormycosis (22). Whether combination anti-fungal therapy is superior to single anti-fungal therapy for mucormycosis remains controversial (23). In addition, there is currently no standardized protocol for evaluating the efficacy and duration of treatment with amphotericin B. The primary criteria currently used are based on the degree of immunosuppression and the remission of symptoms and signs. It is imperative to identify the optimal prevention and treatment strategies for mucormycosis to mitigate its morbidity and mortality rates.

In conclusion, mucormycosis is characterized by rapid progression, high mortality, and a challenging diagnosis. The successful management of our case emphasizes the importance of maintaining a high index of suspicion, timely diagnosis, and prompt initiation of specific anti-fungal therapy. This case report highlights the importance of mNGS and CEUS in diagnosing splenic mucormycosis. Surgical debridement will remain a cornerstone in the treatment of mucormycosis. Treatment strategies for mucormycosis are clear and well-defined in the literature and guidelines. No survival benefit has been shown with combination therapy. What remains unclear is treatment duration, which depends on clinical improvement and the degree of immunosuppression, in addition to the lack of follow-up markers.

## Data availability statement

The original contributions presented in the study are included in the article/supplementary material. Further inquiries can be directed to the corresponding author.

## Ethics statement

Studies involving human participants were reviewed and approved by the Center for Ethics in the First Hospital of Lanzhou University. Written informed consent was obtained from the individual for the publication of any potentially identifiable images or data included in this article.​

## Author contributions

XP: analyzed the patient data and drafted the manuscript. ZW: made significant contributions to the collection of patient data. LW: made significant contributions to the analysis of the patient data. JC: designed the case report and revised the manuscript. All authors contributed to the article and approved the submitted version.

## References

[1] Sung AH Martin S Phan B Benigno M Stephens J Aram JA. Risk factors in people with mold infections that have spread to different parts of the body: A plain language summary. Future Microbiol (2022) 17:1271–5. doi: 10.2217/fmb-2022-0144 36043988

[2] Singh AK Singh R Joshi SR Misra A. Mucormycosis in COVID-19: A systematic review of cases reported worldwide and in India. Diabetes Metab Syndr (2021) 15(4):102146. doi: 10.1016/j.dsx.2021.05.019 34192610 PMC8137376

[3] Arbune M Arbune AA Nechifor A Chiscop I Sapira V. Diagnostic and treatment challenges of emergent COVID-associated-mucormycosis: A case report and review of the literature. Antibiotics (Basel) (2022) 12(1):31. doi: 10.3390/antibiotics12010031 36671232 PMC9854657

[4] Hussain MK Ahmed S Khan A Siddiqui AJ Khatoon S Jahan S. Mucormycosis: A hidden mystery of fungal infection, possible diagnosis, treatment and development of new therapeutic agents. Eur J Med Chem (2023) 15(246):115010. doi: 10.1016/j.ejmech.2022.115010 PMC9734071 36566630

[5] Steinbrink JM Miceli MH. Mucormycosis. Infect Dis Clin North Am (2021) 35(2):435–52. doi: 10.1016/j.idc.2021.03.009 PMC10110349 34016285

[6] Pham D Howard-Jones AR Sparks R Stefani M Sivalingam V Halliday CL. Epidemiology, modern diagnostics, and the management of mucorales infections. J Fungi (Basel) (2023) 9(6):659. doi: 10.3390/jof9060659 37367595 PMC10304757

[7] Osaigbovo II Ekeng BE Davies AA Oladele RO. Mucormycosis in Africa: Epidemiology, diagnosis and treatment outcomes. Mycoses (2023) 66(7):555–62. doi: 10.1111/myc.13581 36856432

[8] Lynch JP 3rd Fishbein MC Abtin F Zhanel GG. Part 1: Mucormycosis: prevalence, risk factors, clinical features, and diagnosis. Expert Rev Anti Infect Ther (2023) 21(7):723–36. doi: 10.1080/14787210.2023.2220964 37262298

[9] Darwish RM AlMasri M Al-Masri MM. Mucormycosis: The hidden and forgotten disease. J Appl Microbiol (2022) 132(6):4042–57. doi: 10.1111/jam.15487 35156271

[10] Acosta-España JD Voigt K. Mini review: Risk assessment, clinical manifestation, prediction, and prognosis of mucormycosis: Implications for pathogen- and human-derived biomarkers. Front Microbiol (2022) 20:895989(13). doi: 10.3389/fmicb.2022.895989 PMC9251460 35794908

[11] Hammami F Koubaa M Chakroun A Smaoui F Marrakchi C Hentati N. Survival of an immuno-competent patient from splenic and gastric mucormycosis-case report and review of the literature. J Mycol Med (2021) 31(4):101174. doi: 10.1016/j.mycmed.2021.101174 34274682

[12] Cao F Qian W Ma Y Wu Y Zhong J. Contrast-enhanced imaging features and differentiation of benign and Malignant focal splenic lesions. Clin Imaging (2018) 49:58–64. doi: 10.1016/j.clinimag 29132054

[13] Trenker C Dohse M Metzelder SK Rexin P Mariss J Goerg C. 71-year-old patient with chronic lymphocytic leukemia (CLL) and hypoechoic nodular spleen and liver lesions with fatal outcome: Presentation of mucormycosis in B-mode imaging and contrast-enhanced ultrasound (CEUS). Ultrasound Int Open (2016) 2(3):E100–1. doi: 10.1055/s-0042-106394 PMC5026038 27689177

[14] Wang J Li Y Luo S Zheng H. Rhinocerebral mucormycosis secondary to severe acute pancreatitis and diabetic ketoacidosis: a case report. Diagn Pathol (2021) 16(1):34. doi: 10.1186/s13000-021-01094-3 33882979 PMC8061203

[15] Liu Y Zhang J Han B Du L Shi Z Wang C. Case report: Diagnostic value of metagenomics next generation sequencing in intracranial infection caused by mucor. Front Med (Lausanne) (2021) 8:682758. doi: 10.3389/fmed.2021.682758 34631726 PMC8494775

[16] Lackner N Posch W Lass-Flörl C. Microbiological and molecular diagnosis of mucormycosis: From old to new. Microorganisms (2021) 9(7):1518. doi: 10.3390/microorganisms9071518 34361953 PMC8304313

[17] Millon L Caillot D Berceanu A Bretagne S Lanternier F Morio F. Evaluation of serum mucorales polymerase chain reaction (PCR) for the diagnosis of mucormycoses: The MODIMUCOR prospective trial. Clin Infect Dis (2022) 75(5):777–85. doi: 10.1093/cid/ciab1066 34986227

[18] Sun Y Li H Chen J Ma Z Han P Liu Y. Case report: Metagenomics next-generation sequencing can be performed for the diagnosis of disseminated mucormycosis. Front Med (Lausanne) (2021) 8:675030. doi: 10.3389/fmed.2021.675030 34746163 PMC8568769

[19] Zhang M Lu W Xie D Wang J Xiao X Pu Y. Metagenomic next-generation sequencing for diagnostically challenging mucormycosis in patients with hematological Malignancies. Infect Drug Resist (2022) 15:7509–17. doi: 10.2147/IDR.S393201 PMC9784388 36570711

[20] Smith C Lee SC. Current treatments against mucormycosis and future directions. PLoS Pathog (2022) 18(10):e1010858. doi: 10.1371/journal.ppat.1010858 36227854 PMC9560507

[21] Meena DS Kumar D Bohra GK. Combination therapy in Mucormycosis: Current evidence from the world literature, a mini review. J Mycol Med (2023) 33(1):101332. doi: 10.1016/j.mycmed.2022.101332 36270213 PMC9472709

[22] Cornely OA Alastruey-Izquierdo A Arenz D Chen SCA Dannaoui E Hochhegger B. Global guideline for the diagnosis and management of mucormycosis: an initiative of the European Confederation of Medical Mycology in cooperation with the Mycoses Study Group Education and Research Consortium. Lancet Infect Dis (2019) 19(12):e405–21. doi: 10.1016/S1473-3099(19)30312-3 PMC8559573 31699664

[23] Lamoth F. The unresolved issues in the management of mucormycosis. Eur J Intern Med (2022) 100:29–30. doi: 10.1016/j.ejim.2022.03.026 35351351

